# Promoting Social Connection and Deepening Relations Among Older Adults: Design and Qualitative Evaluation of Media Parcels

**DOI:** 10.2196/14112

**Published:** 2019-10-03

**Authors:** Isabela Zaine, David Mark Frohlich, Kamila Rios Da Hora Rodrigues, Bruna Carolina Rodrigues Cunha, Alex Fernando Orlando, Leonardo Fernandes Scalco, Maria Da Graça Campos Pimentel

**Affiliations:** 1 University of São Paulo Institute of Mathematics and Computer Sciences São Carlos - SP Brazil; 2 University of Surrey Department of Music and Media Guildford United Kingdom; 3 Federal University of São Carlos Department of Computer Sciences São Carlos Brazil; 4 Federal Institute of Education, Science and Technology of São Paulo Capivari Brazil

**Keywords:** social interaction, interpersonal relations, communication, intervention, experience sampling, mobile apps, photography, video-audio media, elderly

## Abstract

**Background:**

Being socially connected is related to well-being, and one way of avoiding social isolation is to deepen existing relationships. Even though existing relationships can be reinforced by regular and meaningful communication, state-of-the-art communication technologies alone do not increase the quality of social connections. Thus, there is a need for the involvement of a trained human facilitator in a network of older adults, preferably for a short period, to promote the deepening of their relationships.

**Objective:**

This study aimed to evaluate the hypothesis that a human-facilitated, media-sharing social networking system can improve social connection in a small group of older people, who are more vulnerable to social isolation than most, and deepen their relationships over a period of a few weeks.

**Methods:**

We conducted the design and evaluation of *Media Parcels*, a novel human-facilitated social networking system. *Media Parcels* is based on the metaphor of a facilitator collecting and delivering parcels in the physical mail. Extending the metaphor, the system supports a facilitator in designing time-based dialogue requesting parcels from participants that bring out their memories and feelings, in collecting the parcels, wrapping them in annotations that communicate the corresponding requests, and delivering the wrapped parcel to a target person. Qualitative evaluation was carried out in two trials with a group of three people each, one with family members (children and father; aged 55, 56, and 82 years old) and the other with a group of friends (aged 72, 72, and 74 years old), over two weeks. In each trial, data were collected in three interviews (pre-, mid-, and posttrial) and via system logging.

**Results:**

Collected data indicate positive social effects for deepening and developing relationships. The parcel metaphor was easily understood and the computational system was readily adopted. Preferences with regard to media production or consumption varied among participants. In the family group, children preferred receiving media parcels (because of their sentimental value) to producing them, whereas the father enjoyed both. In the friendship group, preferences varied: one friend enjoyed both producing and receiving, while the other two preferred one over the other. In general, participants reported a preference for the production of items of a certain type depending on the associated content. Apart from having a strong engagement with the system, participants reported feeling closer to each other than usual.

**Conclusions:**

For both groups, *Media Parcels* was effective in promoting media sharing and social connections, resulting in the deepening of existing relationships. Its design informs researchers who are attempting to promote social connection in older adults.

## Introduction

### Background

The concept of connectedness is multidimensional and there are multiple definitions and types of connectedness, including social connectedness, that focus on interpersonal relationships. Social connectedness, as defined by Lee and Robbins [1,2], includes feelings of being in a close relationship with the social world, which is critical to one’s sense of belonging and is based on proximal and distal relationships. As social interactions may be transient, feelings of being connected to others may change. Thus, social connectedness may also correspond to short-term experiences of belonging [3]. Having regular and meaningful contact with others usually increases social connectedness, but this type of contact may be hindering for older people because of mobility and geographic constraints.

The global population aged 60 years old or above is growing at a rate of about 3% per year, which is faster than all younger age groups. At present, in Europe 25% of the population is already aged above 60 years old, and in Latin America they account for 12% of the population [4]. This shift in the world’s demographics has led to a need for action across multiple sectors to enable older people to age well and remain a resource to their families, communities, and economies. Thus, successful ageing and healthy ageing have been topics of interest in some of the most recent studies conducted among the older population. The World Health Organization [5] defines successful ageing as the process of developing and maintaining the functional ability that enables well-being in older age. By including well-being in this definition, it goes beyond physical health as it includes domains such as happiness, satisfaction, fulfillment, and feelings of belongingness.

Older people often emphasize the role of social integration and well-being associated with successful ageing [6]. As they age, they wish to maintain their identities and social roles, have relationships, remain autonomous, feel safe, feel like they still have the potential for personal growth, and be able to enjoy life [7-9].

Older adults are more vulnerable to social isolation than the rest of the population. Typically, their social networks shrink with age because of bereavement, relocation, and reduced mobility [10]. Moreover, older adults who are socially isolated have been shown to be more depressed and disabled, be in poorer health, and report lower levels of well-being than those who are socially connected [11]. Thus, it is important that they are able to maintain and deepen social connections with their remaining family and friends and to establish new ones.

Maintaining regular social connections can be challenging for older people living alone, who may struggle to travel to meet distant contacts in person or attend in-person social events. Web-based contact seems to be an obvious solution, particularly given the rise in internet access via mobile devices for adults aged 65 years or older in the United Kingdom [12], Brazil [13], and the United States [14], among others. Such technology can support older adults [15] who prefer traditional one-to-one channels of communication [16,17].

Social connectedness can be stimulated by experiences that remind people of their social relationships. For example, looking at a photograph or listening to a song may remind them of a loved one and their relationship with them. Thus, feelings of social connectedness do not necessarily require physical proximity. Based on these insights, our hypothesis is that a human-facilitated, media-sharing social networking system can improve social connection in older people and deepen their relationships upon deployment over a few weeks.

To evaluate our hypothesis, we designed a new type of social networking system called *Media Parcels* to address social connection in older people and deepen their relationships over a few weeks with the mediation of a human facilitator. Media collection and sharing, orchestrated by the facilitator, lies at the heart of the system, as this activity is meant to stimulate user reflection about current relationships, both strong and weak. In the *Media Parcels* system, parcels of media are solicited by the facilitator for later consumption by specific recipients, with the types of media requested designed to encourage reciprocal intimacy and self-disclosure between an older person and selected social contacts.

Both the intervention duration and the group size are important in the proposed design. One of the main reasons is that a large group size would imply developing a large number of relationships, which is difficult to achieve in a short period and challenging for a facilitator to coordinate. Second, a duration of a few weeks allows the involvement of the facilitator to be short term, and finally, this would allow the study to take advantage of the temporary novelty effect observed with new technology adoption.

In the current design of the system, media collection and distribution are done by a human facilitator among 3 users. In future designs, the facilitator might be supported or eventually replaced by an algorithm designed to allow larger networks.

### Social Networking and Older People

Much research exists on the value of sharing digital media and other physical memorabilia for maintaining relationships but not the honesty and intent [18-21]. Here, we will concentrate on more recent broad social media services and studies of media sharing through social networking systems, especially those targeted at the older population.

Facebook use is now part of daily life for many people around the globe, and a huge number of posts made by friends are delivered continuously to users. An early study on self-disclosure on Facebook by Park et al [22] with 317 student participants showed that the number of and positivity of posts is associated with the intimacy of the relationship, but not the honesty and intent. In a later study with 243 participants, Orben and Dunbar [23] investigated how passive consumption of personal disclosures affects the development of relationships. Passive consumption occurs when a person reads the posts of others without directly interacting with them. Their results suggest that reading high intimacy, self-disclosures increases relationship closeness, triggering similar relationship formation in real-life interactions.

When focused especially on the elderly population, the use of social media is slightly different to other groups. Rebelo [24] conducted an exploratory study in Portugal with 4 older adults to understand the motivations and interests of the elderly population when using social networks (in particular Facebook). The researcher found that, for this particular group, motivation was mainly related to solitude, belonging, reunions, and willingness to improve intergenerational relationships. Regarding shared content, the elderly stated that they like discussing memories but were concerned about privacy, and they thought that most of the published content on the social network site was inadequate or uninteresting. Chakraborty et al [25] studied the privacy-preserving behaviors of older Facebook users in direct contrast to their self-disclosure. They analyzed the profile information and privacy settings of 134 Facebook users aged above 55 years, together with 50 of their friends, and they observed that older adults hide or share information on their profiles depending on what information their contacts share. They tend to be more conservative about information sharing, in line with the findings of Lindley et al [16] and Pedell et al [17].

Sinclair and Grieve [26], in turn, investigated whether older adults could derive social connectedness from Facebook and whether the levels of social connectedness were similar to those seen in younger samples. The analysis revealed that Facebook social connectedness emerged as a separate factor to offline social connectedness, with correlations between the factors indicating that they were distinct constructs. The participants reported levels of Facebook-derived social connectedness similar to those seen in younger samples. About the social effects and benefits caused by the networks, Quinn [27] examined the engagement among novice social media users, aged 65 years and older, in 4 cognitive domains: attention, processing speed, working memory, and inhibitory control. Results reported include the improvement of intervention participants in inhibitory control. Quinn argued that the findings demonstrated that the benefits of social media use at older ages extended beyond mere social engagement and into other domains of everyday well-being.

Chen and Schlz [28] conducted a systematic review to verify the effect of information communication technology on elderly social isolation. Their findings suggested that although the technology lessened social isolation, the technology alone does not guarantee quality of communication among older adults. Furthermore, when communication is not reciprocal, technology use may increase social isolation [29].

Several novel media sharing systems have been designed for the older population, with differing levels of effects on relationships. A number of these systems use situated displays in the home to share materials at a distance, such as photographs, text messages and broadcast media [30-33]. For instance, Garattini et al [34] designed a system to promote opportunistic social interaction among elderly people. Using a tablet, the system broadcast news of different topics and presents functionalities to enable group conversations through voice and text. The researchers conducted a 10-week study with 19 elders and some of their friends and family members. The results suggested that although the broadcasts encouraged social interactions, the quality of the engagement was limited by the absence of an approach to share personal information to help users become familiar with one another.

Waycott et al [31] developed a tool to facilitate message and media sharing and conducted a study in which caregivers used the tool to communicate with their elderly clients. Their results showed that photographs with captions were able to increase and enhance communication, and were well-suited to promote psychosocial care. Similarly, Abrahão et al [35] conducted a mobile digital storytelling study in a care home for older people. The creation of the stories was facilitated by either a formal or informal carer and focused mainly on the resident, capturing aspects of everyday life such as visits, social events, therapy sessions, and health reports. The results showed that the technology stimulated expressivity and creativity in the resident, as well as richer conversations between the resident and other people.

Cornejo et al [32] developed a system geared toward enhancing older people’s offline interactions with their family. The results emphasized how information shared on social media could provide conversational context for the elderly, prevent isolation, and increase offline conversations.

Most studies report positive benefits for maintaining or making new relationships through lightweight multimedia messages. Given the established relationship between the level of self-disclosure and relationship closeness both online and offline, there is an opportunity to explicitly encourage media exchange related to the existing relationship. By encouraging self-disclosure, we mean to encourage participants to talk about feelings and emotions, to talk about topics they do not regularly talk about (eg, intimate things), and to talk about themselves and their relationship toward each other so that using an app might be easier than saying it face-to-face.

## Methods

### Media Parcels Design

*Media Parcels* is a novel social networking system designed to promote facilitated media exchange between users. The interaction underlying *Media Parcels* is based on the metaphor of parcel delivery in the physical mail. First, a facilitator, upon specific requests to participants, collects media and wraps them in text commentary, bringing out their memories and meaning. Next, the facilitator passes the wrapped media parcel to a target person, who in turn unwraps them.

In the general case, Media Parcels supports facilitators in orchestrating interactions among any number of users by intervening in 2 steps: in phase 1 by requesting media parcels from a user and, in phase 2 by sending the parcels to any number of (other) users.

**Figure 1:**
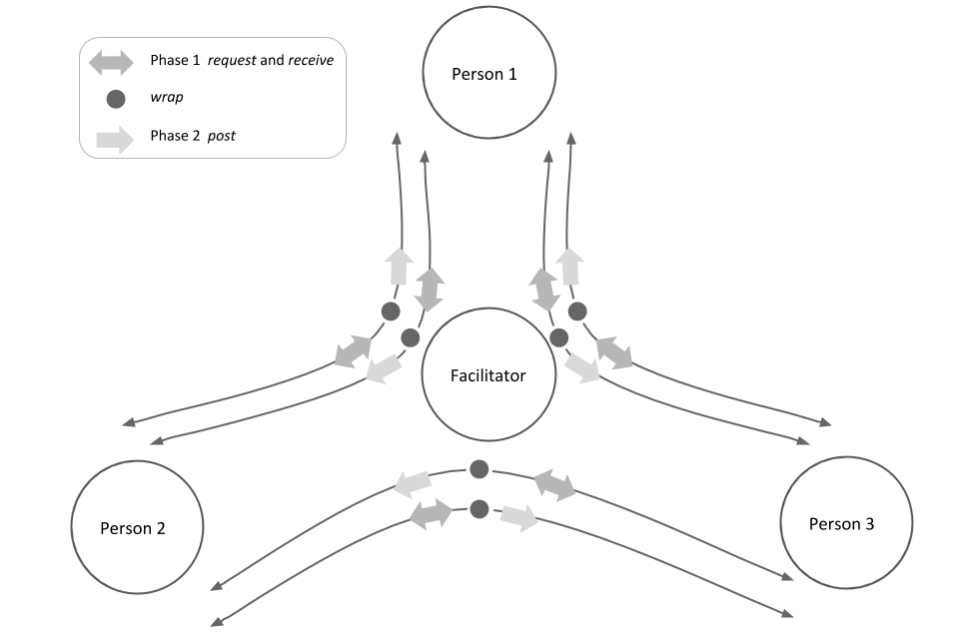
Media Parcels exchange among three persons (P1-P3) in a two-phase interaction orchestrated by a Facilitator, who “wraps” the parcel by adding commentaries.

The particular case of deploying Media Parcels for the balanced interaction among 3 users (P1, P2, and P3) orchestrated by a facilitator is illustrated in Figure 1. For the same scenario, Table 1 details the request of parcels of media (from-to).

As currently designed, *Media Parcels* relies on a facilitator in charge of managing the interaction for the duration of the intervention. In a nonfacilitated approach, *Media Parcels* could, in principle, pass parcels of media between multiple providers and recipients of media, at various times, *ad infinitum*.

In contrast to most current social media systems that rely on the spontaneous posting of media for feedback, the *Media Parcels* approach is based on directed requests for media items which are then shared within a group.

For the deployment of *Media Parcels*, we used the Experience Sampling and Programmed Intervention Method (ESPIM) and an associated platform that support specialists (eg, health professionals) in communicating with their users at particular times of the day via a mobile app [36,37]. ESPIM is inspired by the experience sampling method for collecting self-reports in psychology proposed by Csikszentmihalyi et al [38], combined with the concept of programmed instruction [39]. The ESPIM software platform was designed to support health care and learning interventions in their natural environments throughout the day [36]. Such interventions can comprise open-ended and multiple choice questions, media requests, and deliveries. While creating the interventions, the professional defines the time-based moments in which they should be prompted on the user’s device [40]. 

**Table 1:** Operations for *Media Parcels* exchange among persons P1, P2, and P3 in request-wrap-post interaction orchestrated by a facilitator.

Phase and facilitator (role)		P1	P2	P3
**Phase 1**				
	Media parcel (Request)	from (P1) to (P2)	from (P2) to (P1)	from (P3) to (P1)
	Media parcel (Receive)	from (P1) to (P3)	from (P2) to (P3)	from (P3) to (P2)
**Wrapping**				
	Media parcel (Wrap)	—^a^	—	—
**Phase 2**				
	Media parcel (Post)	from (P2) to (P1)	from (P1) to (P2)	from (P1) to (P3)
	Media parcel (Post)	from (P3) to (P1)	from (P3) to (P2)	from (P2) to (P3)

^a^Not applicable.

### Approach Toward Social Connection and Deepening Relations

Aiming at supporting a facilitator who promotes social connection and deepening of relationships in a small network, comprising older persons and their social contacts, content exchanged via Media Parcels should encourage self-disclosure in the scope of the social relations involved while ensuring both intimacy and privacy.

#### Facilitator

The role of the facilitator includes: (1) designing media requests associated with feelings that encourage self-disclosure and reflection over a specific social contact; (2) wrapping together the media elements received and the associated feelings, without editing them; and (3) mediating the interaction between a pair of users by sharing the content produced between both ends of the targeted relationship.

For the purposes of this study, such a role demands professional skills, so in the case of the two trials described here the facilitator had a background in clinical psychology.

##### Media Requests

Requests for media were deliberately designed to be thought provoking and vehicles for self-reflection and disclosure. For example, the following requests and questions were used, among others, to elicit responses across a variety of media forms, personalized by name: 

- Take a picture of an object that is special to you and explain why (image and text).
- Record a short audio clip of yourself singing a snippet of a song you like and explain why you like it (audio and text).
- Record a short audio to X, saying what he or she means to you (audio).
- What do you like to do with X? Why? (text).
- Send me a picture of a present X has sent you in the past. What was the occasion? How did you feel about it? (image and text).
- Record a short video of you saying something X often says (video).

Media requests were inspired by methods used in design and behavioral psychotherapy. We used two strategies for the design of media requests, one concerning the type of media, and the other, the media content. Different types of media may produce different emotional responses [41], so we designed media requests to be balanced across image, audio, text, and video to provide participants with all types of media once, and regarding media content, the media requests were designed to probe participants’ relationships and themselves. 

Behavioral psychological interventions make use of asking questions, making requests, and giving instructions or suggestions for clients to carry out specific or generic actions outside the therapeutic setting [42]. Asking questions about happenings or feelings serves to gather information and also promotes self-observation, self-reflection, and self-knowledge. In our study, the media requests and questions about participants’ feelings were designed to encourage all participants to reflect on their relationships and express their feelings toward one another by producing media with emotional content. Furthermore, specifically for the older participants, the questions and requests also encouraged reminiscence of precious moments and self-disclosure.

The questions and requests might also be referred to as relationship probes, as they have the character of cultural probe questions for self-report except they are focused on personal traits and relationships rather than culture [43]. Cultural probes is a technique used to inspire ideas in a design process. Typically, the probes are small packages that can include any sort of artifact along with evocative tasks that allow participants to keep record of specific events, feelings, or interactions. It serves as a means of gathering inspirational data about people’s lives, values, and thoughts.

##### Media Wrapping and Sharing

**Figure 2:**
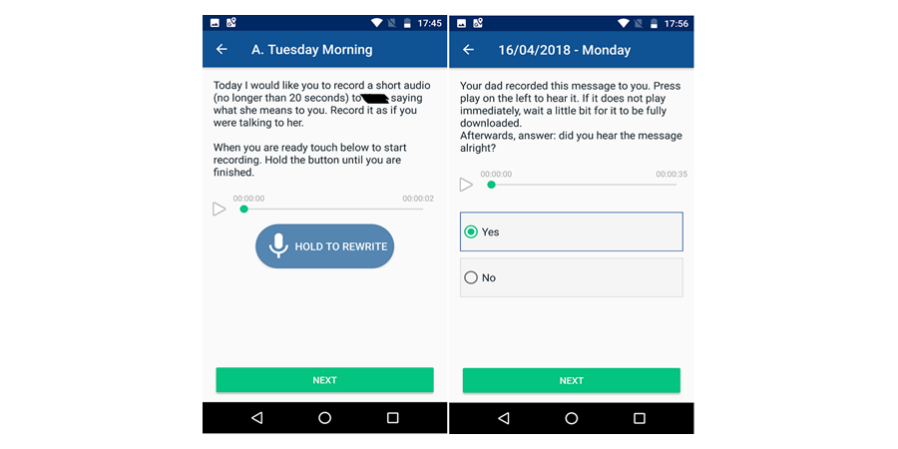
Typical media request and delivery screens. Left: media request in phase 1 to the older adult. Right: media delivered to another participant in phase 2.

For each media item collected, before sending the item to the target person the facilitator included a text comment to expose the request that created it. An example is given in Figure 2, with the facilitator requesting for one person to record a piece of audio for another person saying what she means to you. When sharing that media, the facilitator includes “Your dad recorded this message to you”. As indicated by this example, no editing relative to the media collected took place.

#### Older Person’s Social Contacts

Social contacts of an older person may be inter- or intragenerational, such as younger family members (their children or grandchildren) for intergenerational contacts, and the same generation friends or family members for intragenerational contacts. According to Lindley et al [16], the dynamics of family and friend relationships are very different, especially in terms of expected social support. Considering the two different types of social contacts, we designed two trials devoted to each type of relationship: one older person with two younger family members, and three same-generation older individuals that were friends.

For practical reasons related to our international collaboration, we conducted the first trial with family participants in the United Kingdom and the second trial with friendship participants in Brazil. The system and methods for both trials were largely the same and are reported in study 1 for the UK context, with variations outlined in study 2 for the Brazilian context.

The studies reported in this paper were designed so that phases 1 and 2 lasted around one week each. This amounted to a fixed period intervention aimed at increasing social connection between these partners. We designed a media collection phase (phase 1) and a media sharing phase (phase 2), so that participants could clearly discriminate between producing and receiving personal media content. In both studies, participants were aware of the two distinct phases and that the produced media in phase 1 would not be immediately shared with the other participants. They were also informed that the human facilitator, who was a clinical psychologist, would be in charge of sharing the produced media and its associated feelings from phase 1 with the other participants on phase 2, without editing the content. Participants were also aware that, once the facilitator mediated the media sharing, this person would have access to the content of the media and its associated feelings. In addition, the participants were informed that, after phase 2, the facilitator would not mediate their interaction anymore; thus, if they wished to talk about the media content received, they should use their conventional communication channels.

### Study 1: Family Trial

This study focused on supporting an older person through media exchanges with two younger family members. The exchange of media parcels was asymmetric because they were not collected and delivered between the younger participants.

**Figure 3:**
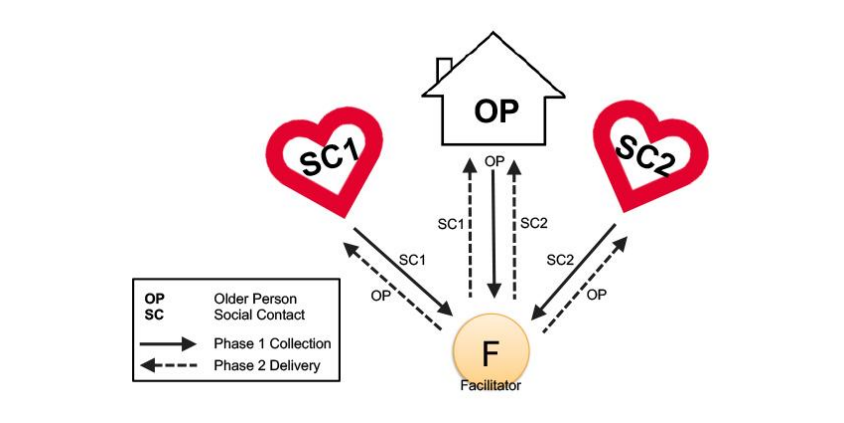
Network configuration and media flow in the Media Parcels system for study 1.

Figure 3 shows the network configuration for study 1. An older adult who lives alone exchanged media (text, audio, image, and video) with 2 other social contacts. The facilitator (the first author) orchestrated the exchange in 2 phases. In phase 1, lasting a week, media relating to each pairwise relationship with the older person was collected by the facilitator at regular intervals. In phase 2, also lasting a week, that media was distributed to the reciprocal partner in each pair at regular intervals. As the older person is linked with 2 people, while those people are only linked to one and not each other, we collected and distributed twice as much content from the older person as from the reciprocal partners, as shown by the arrows in Figure 3.

#### Media

A typical media collection and sharing screen is shown in Figure 2, as previously presented. Requests for media were deliberately designed to be thought provoking and vehicles for self-reflection and disclosure. For example, the following requests and questions were used, among others, to elicit responses across a variety of media forms, personalized by name:

- Take a picture of an object that is special to you and explain why (image and text).
- Record a short audio clip of yourself singing a snippet of a song you like and explain why you like it (audio and text).
- Record a short audio to X, saying what he or she means to you (audio).
- What do you like to do with X? Why? (text).
- Send me a picture of a present X has sent you in the past. What was the occasion? How did you feel about it? (image and text).
- Record a short video of you saying something X often says (video).

#### Participants

Recruitment requirements for the older person were as follows: (1) aged 60 years or older; (2) lives alone; (3) is based in the Guildford area, United Kingdom; and (4) is able to nominate at least two other social contacts who are able to participate. All names were changed to preserve participants’ identities. We formally broadcasted the search for participants for 6 weeks through the University of Surrey academic email to the existing network of research groups and personal contacts, on the research group website, and by partnering with Age UK Surrey and the University of the Third Age, Guildford. The participants recruited were resultant of personal contact.

Participants were 1 older adult, Paul (aged 82 years), and 2 of his children: Karen (aged 55 years) and Charles (aged 56 years). Paul chose them because he considered them relevant people for social support in his life. Paul is an electrical and electronic engineer and still works part-time, mentoring engineers at a company. He did not have any cognitive impairment according to the Mini-Mental State Examination to measure cognitive health [44]. He was also living alone for the past 6 months and feeling more isolated since his wife passed away. Charles, his son, lives close to him. His daughter, Karen, lives in Kenya, Africa, but was visiting her father for the first week of the trial. The field data collection was done from late April 2018 to late May 2018.

#### Pretrial Interview

A pretrial semistructured interview was conducted face-to-face with each participant. With the older person, we enquired about his relationship with his two children. To access feelings of a specific social connection at the individual level between Paul and his children, we designed a Relationship Semantic Differential Scale (RSDS, Multimedia Appendix 1) with 16 pairs of contrasting attributes related to social relationships. Participants were instructed to express their agreement with the attributes on a 7-point scale. As typical of semantic differential scales, the closer the participant responds to one of the attributes the more they agree with it [45]. Paul was asked to rate his relationship with each child separately. Finally, the *Media Parcels* mobile app was downloaded and installed on a dedicated tablet. Paul was given this tablet and trained in how to use it. This took a total of about 1 hour and 30 min.

Each of Paul’s children also answered a similar semistructured interview enquiring about his or her relationship with their father and responded to the RSDS. The *Media Parcels* mobile app was downloaded to their existing smartphones and they were trained in how to use it. This took about 30 min per person.

#### Trial Period

Following the pretrial interview, the system was activated on all 3 devices and proceeded to collect media parcels from participants over 7 consecutive days in the first week of the trial. These were analyzed and selected by the facilitator for redistribution over the following 8 consecutive days of the second week for the social contacts and 9 consecutive days for the older person. The amount of media parcels received in the second week was based on the amount of relevant media parcels produced by participants in the first week; therefore, it varied among participants. The older person, Paul, received 3 collection messages or 1 delivery message from the system each day, whereas his children Karen and Charles received only 1 collection or 1 delivery message a day. Notifications of each message sounded and appeared on the receiving devices, but users were able to defer answering or reading them until later. Users could also choose to decline answering any request. Once a media delivery had been seen, users could not directly respond with feedback to the person who sent the media, as is usual on most social media platforms. It was a design decision not to support interactional closure to encourage participants to contact each other offline or using their existing preferred communication channels. This decision was based on the fact that new technology solutions on communication mostly force users to abandon their usual channels to adopt new ones. This can be aversive to those people who take more time learning to use a novel technology, which is the case for most of the older population [46].

#### Midtrial Interview

At the end of the first week (phase 1) of the trial, we conducted a midtrial interview. Participants once again each responded to the RSDS and were interviewed about the feelings generated by producing materials about their relationship. Participants were also briefed about the delivery of phase 2 of the study.

#### Posttrial Interview

After phase 2, we conducted final semistructured interviews with all participants, separately, about their relationships, connectedness toward one another, and experience using the system. They then responded to the RSDS. The meetings lasted an average of 30 min for each social contact and 1 hour for the older person. In this last interview, they also evaluated their experience with the ESPIM mobile app by answering a User Experience Questionnaire (UEQ) [47] and a System Usability Scale (SUS) [48]. All interviews were conducted in the participants’ native language, English.

### Study 2: Friendship Trial

To test out the value of the system for deepening relationships with same generation friends, we recruited 3 older people with different levels of acquaintance, as described below. We decided to treat all these participants equally in the trial by using symmetrical media sharing between all three, where P1, P2, and P3 are three elderly ladies. This means the scripted dialogue between participants and the facilitator implies that media were collected in phase 1 from each participant about their other 2 partners and distributed to those partners in phase 2.

#### Participants

Recruitment requirements were as follows: (1) aged 60 years or older; (2) lives alone; (3) is based in São Carlos, Brazil; and (4) is able to nominate at least two other same-generation social contacts to also participate. The research was broadcast in a digital literacy course for older adults. In total, three elderly ladies volunteered. They described themselves as friends or acquaintances. Ronda is a 72-year-old retired teacher who was divorced and had a son. Linda is also aged 72 years and is a retired administrative assistant) who was never married and has had a boyfriend for the past 8 years. Finally, Irene is 74 and a retired laboratory assistant. All participants were Brazilian. They had some experience dealing with smartphones and tablets. None of them had any cognitive impairments.

**Figure 4:**
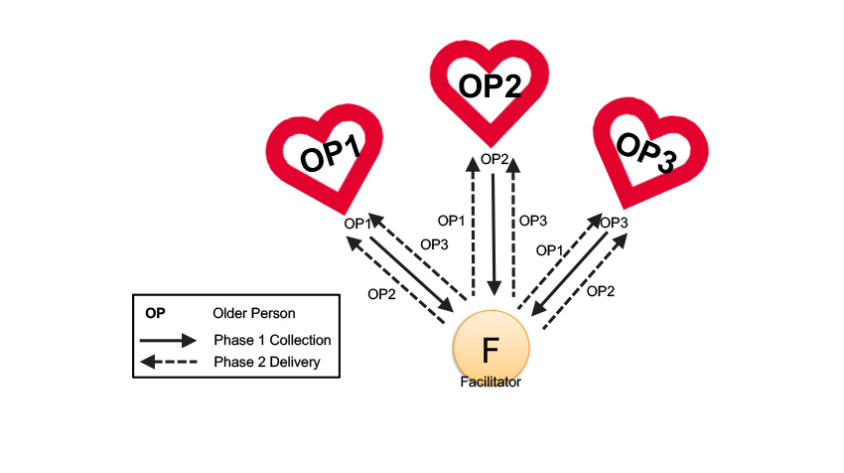
Network configuration and media flow in the Media Parcels system for study 2.

The network configuration and media flow in study 2 is presented in Figure 4. Both Ronda and Linda were more connected to Irene, who acted as a friendship mediator between the two others. Ronda and Linda were friends for about 10 years and they both described being close to each other. Linda and Irene have been friends for over 30 years. They worked together, and before their retirement they were very close. Irene said their relationship started to go cold after a while, especially after Linda started dating. Ronda and Linda consider themselves to be friends, they have known each other for 10 years, though they do not call or arrange to meet in person as this is always mediated by Irene. In addition, most of the time Irene is the one who initiates social contact with both friends. None of them described themselves as socially isolated, but Ronda expressed interest in deepening her relationship with both her friends, Linda and Irene would like to revive their friendship that had become distant over the years, and Linda would also like to become closer to Ronda.

#### Procedure

The procedure was identical to study 1: (1) pretrial interview; (2) media collection; (3) midtrial interview; (4) media sharing; and (5) posttrial interview. In this case, the interviews were conducted in Portuguese. The total length of the data collection, from pre- to posttrial, was 20 days. Media were shared symmetrically as described above. The media requests were specially tailored to suit the particularities of the three participants and to deepen their relations. As before, requests were to share information about themselves (personal requests) and about their relationships with each other (relationship requests).

In phase 1, a total of 14 requests were designed for each participant: 7 personal and 7 about the relationships. The requests involved the same media types as in study 1 (text, audio, video, and image) as well as text commenting about their feelings toward what they had produced. Among the images requested, there were photos of people (friends participating in the study), objects (presents they received from each other, special objects, and objects that reminded them of each other), and places (favorite spot in the house). The audio requests were to share a memory from childhood, talk about a miracle in their lives, and record a song snippet. The video requests were to record an ongoing activity, talk about a good day they spent with the friends, and record a video message to the other friends. The text requests were to write about what they would like to do more with the friends and to write about their favorite singers, television shows, and activities. A total of two requests were sent out each day, one in the morning and another in the afternoon for 7 consecutive days. As in study 1, participants could respond to the requests immediately after they received the sound reminder or any other time during the day. In phase 2, we shared the most compelling media via the system, which, in the case of this study, were the media and associated feelings with more emotional and personal content. All participants received 1 media parcel per day for 9 consecutive days.

## Results

### Study 1: Family Trial

#### User Experience, Engagement, and Patterns of Interaction

The results of the user experience and usability rating scales were very positive, with the SUS results indicating an average score of 77.5 points. This is about 10 points higher than average, and a B+ on a scale ranging from A to F. The UEQ scores were at excellent or above average across all dimensions. Overall, participants were able to use the app without any issue other than difficulty in identifying the status of video recording when the Android video app is used, an issue not under the control of the designers. Such positive results, which relate to the attention given to guidelines during the design of the mobile app [40], contrast with the poor accessibility of many apps when used by older people [49,50]. Looking ahead, the experience and usability scores were very similar for study 2 (average SUS=79.17 and slightly higher UEQ scores). Therefore, the rest of the paper concentrates on the process of using the system and its value for relationships.

In phase 1, the media collection phase, all participants responded to more than half the media requests. Paul responded to 76% (16/21) of media requests on a daily basis. He interacted with the app immediately after the sound reminder in about 38% (8/21) of cases, and preferred to use the app when he had time to engage in the requests:

I heard the alarm, but if I was busy at that moment, I just ignored it (...). I knew I had to do the tasks, so I tried to come back to it when I had time.

Paul

On average, he spent 5 min per interaction, although he spent more time on the requests that required him to retrieve materials, such as pictures, written material, and objects.

Karen responded to 100% (7/7) of media requests. She interacted with the app on two separate days and preferred to respond to most of the requests in a row. It took her an average of 3 min per interaction. She never responded to the requests immediately after the sound reminder:

I preferred to do it all at once, in a day I had time.

Karen

Charles presented the lowest rate of engagement, 55% (5/9). In the intermediate interview, he said he did not interact with the app as much because he had a busy week. He spent 10 min on average in each request and also preferred to interact with the system when he had time, despite the sound reminders.

In phase 2, the media sharing phase, the engagement with the app was higher. Paul and Karen interacted with all of the media shared and Charles did not view only one of the parcels of media, interacting with 87% of them (7/8). The pattern of interaction with the app also changed in phase 2. This time, Karen and Charles interacted with the app almost daily and usually before the sound alert was triggered as a reminder. This suggests an expectation to receive the media produced by the father, which was confirmed in the posttrial interviews in which they reported being curious about what they would receive that day.

#### Type of Media and Media Content

The requests were effective in leading to self-disclosures revealing user’s personalities, memories, and relationships. For example, a response to a request for a picture and explanation of a special object by the older person is shown in Figure 5. The story of this object is touching and something Paul may not have posted spontaneously on a social media site.

On the basis of their experiences producing and receiving media, we asked participants about their favorite types of media and media content in each phase of the study. For Paul, the most important feature was the content of the media and not the type itself. Thus, when producing media, his least favorite was typing (text) “It was boring, I am slow (...) but not impossible.” Regarding the type of media received, he said that one of his favorites was a song snippet from his daughter singing (“You are my sunshine”). He said that this media inspired him to get back to learning the Ukulele, so that he could play that song for her in the future. Charles enjoyed more producing videos because he thought it was an opportunity for him to show something he was proud of. He also reported that he would have enjoyed recording longer videos. He enjoyed receiving the photos and videos best because they were visual. As for Karen, she enjoyed producing all types of media and was pleased that she had the opportunity to do a little bit of everything. She thought that the different types of media made the tasks more enjoyable and less predictable. When she received the media, she enjoyed text, audio, and video more than photos:

The photos were not surprising, they were not new to me (...) the others were an expression of dad in that moment.

Karen

**Figure:**
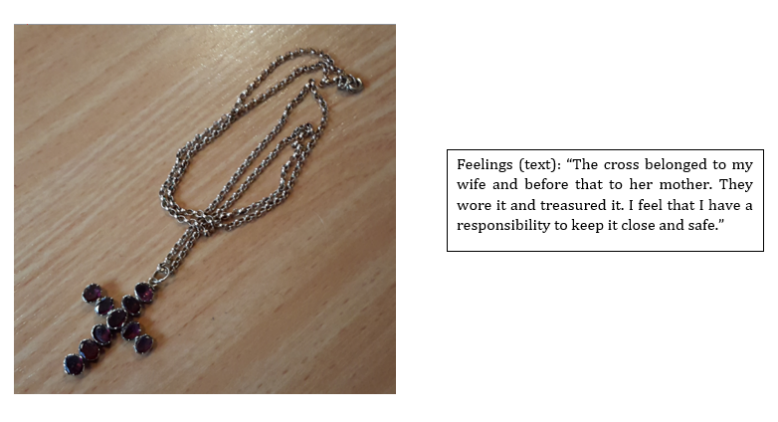
Examples of media collected in phase 1 (image at the left and text at the right).

#### Producing Media Versus Receiving Media

Both Karen and Charles preferred receiving media rather than producing them. They felt good being told good things about themselves by their father and because it was a surprise to see what their father had to say to them. Also, they considered this phase more effortless. Paul also reported that the second week was easier, but he enjoyed both producing and receiving the media:

I enjoyed them (...) it was a good surprise (...) and I was proud of them [Karen and Charles] for participating (...). It was like aconversation.

Paul

#### Feelings of Specific Social Connectedness

All participants reported feeling closer to each other and contacting each other more than usual during the *Media Parcels* trial. This is reflected in the relationship (RSDS) scores taken at the beginning, middle, and end of the trial shown in Multimedia Appendix 2. Scores generally rose in the trial for Paul and Karen. Interestingly, their rise in scores was greatest in the middle of the trial, after media collection but before delivery. This suggests an effect of feeling closer to someone when encouraged to think about them through a media collection task. Charles’ scores remained about the same, perhaps because he found the exercise harder than the other two in terms of expressing his feelings (see Multimedia Appendix 2).

Paul reported that he felt that his contact and relationship with his kids changed during the study:

We ended up being more thoughtful, because of the questions asked. We have become closer and honest to each other.

Paul

He reported that producing and receiving the media and talking about the feelings associated with them made him think about being more effective and also about how to get things done in general:

I realized I was letting things slide. You stimulated my attitude (...) because I had to do the activities it also encouraged me to do other things I needed to do.

Paul

He also thought that doing the activities made him think about his kids much more than usual, and he contacted them more than usual:

It made me think more about emotions and feelings (...) we usually let it go and don’t make time for ourselves. I was happy to do that (...) I was relieved to have a reason to do that.

Paul

Charles reported that, for him, talking about feelings was not easy and as the activities required that he felt as though they were not simple. However, he considered that he thought and contacted his father more than usual during the study (“I did think of him more when I was doing the tasks”) and reported that he felt closer to his father when doing the tasks and when receiving the materials from him. He also pointed out that this type of intervention would have been even more relevant if they were living further apart.

Karen considered that the activities made her think about her father in a different way and remember good moments from the past:

It made me think about my dad in a different way.

Karen

About receiving the media, she reported:

It was nice to see how much I was meaning to Dad (...) he was appreciating things. I felt loved and valued. You got a more intense relationship snapshot from us. It was interesting to see how we were interacting (...). I really enjoyed the way everything was handled. It made me feel closer to dad. It was something that brought me, my dad, and my brother together.

Karen

### Study 2: Friendship Trial

#### Engagement and Patterns of Interaction

In phase 1, there was a high level of engagement with the media requests for all participants. Ronda and Irene responded to 100% (14/14) of the media requests, and Linda to 71% (10/14). Linda did not respond to any of the video requests because of difficulties using the video recording tool in the tablet, despite being trained to do so twice by the researchers. All participants interacted with the system on a daily basis, except for 1 request that Linda completed on the following day.

All participants preferred to interact with the system when they had time, rather than immediately after a request or parcel had arrived. Irene and Linda only responded to the requests immediately after the sound notification in 7% (1/14) of the times, and Ronda in 28% (4/14) of the times. On average, Ronda spent 4 min 23 seconds per interaction, Irene spent 3 min 51 seconds, and Linda spent 5 min 42 seconds. The replies that took the longest were those involving existing materials, such as pictures and objects.

In phase 2, the media sharing phase, the engagement of interaction with the app was 100% (9/9) for all participants. They interacted with the received media parcels on a daily basis. Regarding the sound reminder, Ronda and Irene interacted with the app in the same proportion before, after, or immediately after the reminder 33% (3/9) of the time. Linda interacted with the media parcels immediately after the sound alert 55% (5/9) of the time and before or after it 22% (2/9) of the time. This again reflects increased interest and anticipation in receiving media.

#### Type of Media and Media Content

As in study 1, the media parcels elicited from users were often more intimate than social media posts or even than things they might have said to each other face-to-face. In fact, some had the character of greeting cards that allowed people to express deeper feelings than what they are comfortable saying in person. For example, a request to record a message about her friendship with Ronda caused Irene to say the following:

She is a very kind friend, very caring, very dedicated. I think that regarding my friends I am privileged. Ronda is someone that I like very much.

Irene

When we asked Ronda to say how she felt about this to us, she wrote:

I appreciate the message very much and it made me emotional. I am what I am! I prioritize my friends because they are the shoulders that I lean on. Listening to the audio from Irene I feel safe and cared for. I know I can count on her friendship forever. Thank you, my friend.

Ronda

Regarding their favorite types of media, participants varied. While producing the media, Ronda liked pictures, writing, videos, and audios in that order. Her favorite tasks involved pictures because she retrieved them from old materials that reminded her of stories from the past that she had forgotten. One of her favorite tasks in the media collection phase was to talk about her favorites (singer, television show, and hobbies):

Because of that I started to listen to music that I loved but had forgotten.

Ronda

About the received media parcels, she reported that she did not have a favorite type and that the most important aspect was the content. She appreciated the messages she received from the friends:

I was moved and thought how true friendships are important in our lives. Who has friends has everything!

Ronda

She picked out 2 favorite media parcels: (1) the friends’ childhood stories (“They reminded me of my own childhood. We usually do not share old stories like that”); and (2) their personal profiles. This was a composite parcel made by the researchers. The format was a magazine cover with each participant’s photo and information that they had provided about themselves, in addition to the information provided from the friends to each other. She said that she really related to her own profile and was moved by it. She also thought that the profiles were a good snapshot of each of the friends.

Similar to Ronda, Irene also enjoyed producing the photos:

It was cool. I looked for old, forgotten photos from my friends but also family. It was good to revisit these materials.

Irene

Then her order of preference was audio, video, and text:

I do not like so much to show in pictures, so I did not like very much to show my face in the videos. But the audio was fine. I also don’t like writing (...) I have never been good with the written word.

Irene

Her favorite activity was to record an audio about her childhood:

It made me remember my childhood, the people from my childhood (...) it was a good time of my life.

Irene

While receiving media, she said that she enjoyed everything and that the content was more important than the format. She noted that she especially enjoyed receiving the videos and pictures and that her favorite media parcel was an audio she received from Ronda:

I felt truly touched and even cried (...). I never expect compliments from other people and I don't even know if I am all that (...) I immediately picked up the phone and called her to say how much she means to me

Irene

For Linda, she enjoyed producing the types of media in the following order: text, photos, audio, and video. Although she likes to watch videos, she had difficulties using the device’s camera to record videos by herself, which was frustrating for her. The media she most enjoyed producing was to find a snippet of a song to record:

I did not want to sing, but I enjoyed looking for a song that I liked to record a bit of it.

Linda

About receiving the media, she said that she appreciated all of them, irrespective of type.

#### Producing Media Versus Receiving Media

Feelings about preferring the producing or the receiving of the media parcels were different across participants. Irene preferred to receive the media:

It is more fun (...) you get to know more about the other person and their feelings (...) I felt emotional and even cried over some materials [messages from the friends].

Irene

Receiving the personal histories about the friends also made her realize that they had similar life experiences. Although she also enjoyed going through old photos because they reminded her of good times. Ronda reported that she enjoyed them both:

I loved them both (...) one phase completes the other (...). If you produce something and do not get anything back, you do not get closure. Producing the materials made me rethink my life, good times and difficult ones (...) and how I have overcome the difficulties (...). I visualized myself back in time, it was as if I was reliving it. I really enjoyed it. It is an interesting approach to carry out with my other friends as well.

Ronda

About the media sharing phase, she reported:

I always expected the messages as a surprise (...) the photos and audios moved me.

Ronda

As for Linda, she enjoyed producing the media parcels because she had the opportunity to revisit bits of her life and moments with the friends:

It touched me (...) I got in touch with my emotions, with personal stuff. It was a bit of work having to look for old materials, but it felt good to revive the friendship (...) it was getting cold

Linda

As in study 1, receiving the media in general produced more spontaneous and emotional responses about their feelings.

#### Feelings of Specific Social Connectedness

During the study, all participants reported feeling closer to each other, thinking about each other, and getting in touch more than usual. All participants commented that during the study, they were encouraged to talk about personal things and talk about how they felt about their friendship in a way that they would not naturally do. Irene said:

Sometimes it is hard for me to express certain feelings (...) this helped me to do that.

Irene

Ronda reported:

These talking points do not come to our conversations by chance. The sequence of questions brought memories (...) and the opportunity to express ourselves in a different way (...) the questions helped me to express my feelings.

Ronda

Although she had a stronger bond with Irene, she reported that she got more involved with Linda as well.

Irene reported that she felt closer to the friends in the media collection phase but even more in the media sharing phase. Irene was moved by the messages she received from both friends, and she decided to call them both:

I called Linda and I said, “I do not say it enough but I love you (...).” I also called Ronda to say that she is really important in my life and that I don’t know what I would do without her.

Irene

After this call, Linda also called Irene once, and Irene called both friends to schedule a night out including 3 other friends. In this occasion, Linda spoke about a misunderstanding she had with Irene 10 years earlier that she still felt hurt about. Irene could not go to Linda’s birthday, so she sent a gift through a friend. Linda did not appreciate it because she would rather have Irene come over in person to bring the present. Thus, she returned the gift and never talked about it again until this moment. They said that this was a good opportunity to set things straight and get closer to each other again, and it ended in a group hug. However, Irene felt a little hurt that Linda was still mad after such a long time. Ronda also commented that she and Irene will try to include Linda more in their social gatherings:

Because she has a boyfriend, we do not invite her a lot to go out with us (...) but she really enjoys our company and we will try to include her more.

Ronda

These reported feelings of closeness are reflected in a systematic rise in their RSDS scores, as shown in Multimedia Appendix 3, at the pretrial, midtrial, and posttrial periods. The only drop was between the mid- and posttrial periods for Irene’s feelings of closeness with Linda. These went down because of the conflict over the birthday present reported above. This shows that developing relationships is a complex business. It may result in relationship work that raises contradictory feelings of closeness and distance within the same relationship pair.

## Discussion

### Principal Findings

We designed a novel communication system called *Media Parcels* that makes time-based requests for media and distributes them within a minimal network of just a few social contacts. The social networking system we designed aimed to support older people in deepening their social connections and relationships while still respecting their privacy concerns.

Broadly, our results indicated positive social effects for both deepening and developing of relationships. The elderly participants perceived personal and more intimate social connection as important to their lives, corroborating with other studies that present social integration with family, friends, or community as crucial to well-being in older age [6-9]. Our participants also appreciated the computer-mediated system as a means to encourage self-disclosure and initiate conversations about feelings toward one another, with more deep and meaningful content. This result supports the findings of Lindley et al [51] that explored the attitudes of older adults to keeping in touch with people who are important to them. The authors found that the older adults wanted to be in touch, and that staying connected was worthy of dedication. Moreover, the participants most valued the communication that is personally expressed, which requires a level of intensity that contrasts with the lightweight interaction that is increasingly afforded by new technologies. This was precisely fostered in our study by the *Media Parcels* system, in which social interaction topics were designed to encourage meaningful reflections and expressions of feelings. The importance of fostering relevant, meaningful social interactions is highlighted in studies by Carstensen et al [52,53], which suggest that older people are more motivated to derive emotional meaning from life and establish intimacy with other people, presenting a preference to invest in relationships that are emotionally rewarding and significant to them.

Testing the *Media Parcels* system on a trio of family members and in a separate trio of friends exposed values common to both networks as well as values exclusive to each social group. It also revealed new opportunities for computer-mediated communication.

### Family Connections

The different family members showed differing levels of engagement with the system across media collection and delivery phases. The older person Paul was most enthusiastic overall, and his daughter Karen was a close second. However, his son Charles started out as a skeptic who found it difficult at first to respond to media requests about his father. All three became more enthusiastic in the second phase of the study when they received media parcels relating to Paul. Karen summed up their experience about the system by saying that it brought them all together in a way that would not have happened spontaneously. This is an important observation from the daughter, as the system was configured to focus on her father and his relationship with family following the death of his wife 6 months earlier. It shows that even bilateral media sharing of this kind stimulated reflections on family as a whole and discussion outside the system that affected all relationships. Thus, the system fostered a shift in the content of the communication between the older participant and his children, from basically discussing family affairs and obligations to talking about feelings toward one another and interests. This is particularly relevant as the complex nature of family ties, that includes feelings of both togetherness and responsibilities, may produce negative feelings with greater degrees of obligation and formality associated with familial relationships when compared with friendship [54]. Interestingly, participants told us they rarely discussed the media parcels themselves, in case they ruined the surprise of pending deliveries during the trial. The parcels were also about feelings that participants found hard to express or discuss in person.

### Friendship Relations

Media sharing was made trilateral in the friendship trial, with three older people of differing closeness to each other. Again, we found various levels of engagement and different effects of the system across participants, but a positive endorsement from all of them in the end. In fact, Ronda wrote to us after the trial saying how much she missed the rhythm of the parcel requests and deliveries and its new kind of connection to her friends. There was greater interest in media collection between friends than in the family trial, and all the participants perceived their friendship relations as precious and important to their well-being. In the specific case of the friendship between Ronda and Irene, they even spent time with each other more frequently than with family members and often offered emotional and health-related support to each other, because of either physical distance from close relatives or not desiring to be a source of concern to them. This points out the importance of having friendships later in life and is aligned to other studies that discuss the benefits of having peer relationships on the well-being of older people [55,56]. However, some points of contention were raised by the deliveries. This was most dramatically illustrated in the story of the rejected present between Irene and Linda, which raised a forgotten issue for Irene but resolved it for Linda. This example shows not only the power of the system and particular media requests to access facets of a relationship but also the dangers of doing that sometimes for relationship closeness.

### Facilitation

A total of 2 types of facilitation seemed to be going on in the trials: (1) the media parcels themselves, as designed, were facilitating reflection and communication between parties and triggering further conversation outside the system; and (2) the human facilitator in the loop, with a background in clinical psychology, authored requests and selected responses to maximize positive effects on relationships.

Concerning the media parcels themselves, we found that participants could express feelings to each other that were hard to communicate face-to-face, as in the use of greetings cards. In this respect, media requests were effective relationship probes, revealing aspects of a relationship to other partners as well as to us. Finding and selecting media to share was also motivating, especially for Ronda and Linda in the friendship trial. This caused them to retrieve forgotten images and other materials and remember the good times in the light of intervening events (see also Frohlich et al [57]). These findings are similar to those in a recent study of media sharing to facilitate young people’s conversations with relatives having dementia. The young people were encouraged to find media relating to the person with dementia through a system called *Ticket to Talk* and use these media as a kind of conversational playlist to stimulate conversation [58].

As for the human facilitator in the loop, human facilitation amplified the effect of media exchanges by personalizing them to the participants. It also continued outside the system, as the facilitator also conducted the interviews and became a sounding board for the participants’ reflections on their relationships. In fact, the facilitator, the first author who is a trained clinical psychologist, realized that it could be a powerful tool for counseling. This raises issues for the future of the approach in terms of the levels of facilitation involved. If the intervention lasts longer than a few weeks, how personal should the media be to deepen relationships between particular people? And how important is it to have a professional facilitator designing and monitoring media?

Regarding the first issue, if a system such as *Media Parcels* should be used for longer periods, then the content of the media requests should be balanced with deep personal content and lightweight content, so that the media exchange does not become burdensome.

With respect to the second issue, in our study, the facilitator had a key role in designing the media requests and collecting and distributing the media produced by the participants. It is not uncommon to have human facilitators mediating technology or Web-based social interaction for vulnerable populations. For example, in Abrahão et al [35], the facilitators had a central role in helping the creation of digital storytelling by a care home elderly resident with dementia. Another example is the Scrapbook Circles network that is designed to allow disabled users to post content to friends and family through facilitators, if they wish. Similarly, the *Media Parcels* system could be easily scaled up to dozens of users by expanding the number of facilitators. However, personalized interventions such as the ones reported in this paper have a limit on scaling; a human facilitator dedicated to the task may be able to monitor and mediate from 3 to 4 dozens of groups weekly.

In contrast, if personalization is not a requirement, the system could be scaled up to hundreds and thousands of users using predetermined templates and algorithms, such as those used in recommender systems toward suggesting books and songs. Also, by indicating users’ relationships in the system, it is feasible to create algorithms that automatize content distribution, so received media can be redirected without the need of facilitators. The ESPIM system used to deploy *Media Parcels* already allows the use of predefined templates and automatic media distribution. The figure of the human facilitator could also be removed by adding in the system functionality of direct communication between participants over a predefined set of conversational topics and media sharing requests. Future research could explore both options to reveal more about the role of media in deepening relationships at a distance, especially for the older population.

### Communication

The communication dynamics of the *Media Parcels* system was unusual: (1) it was facilitated by a human who administered a time-based dialogue; (2) the pace of the facilitator-participant dialogue was slow, ranging from 1-3 times a day; (3) the explicit separation of the collection and delivery phases allowed us to assess the effects of each phase; (4) the distinct phases introduced delay into the communication, resulting in media exchange that was neither wholly asynchronous nor wholly synchronous; and (5) the introduction of a delay into the communication resulted in anticipation of media deliveries in which participants met the parcels as they arrived in real time.

Such communication dynamics could be adapted within the current system design. For example, the time-based dialogs could make use of different media requests between different configurations of people within a wider network, interleaved more closely in time with their delivery. Our findings from study 1 support the evolution of an intervention approach that could be offered professionally to individuals as a kind of relationship therapy. Moreover, the findings from study 2 support a self-paced long-term approach of unfacilitated media sharing within small groups. To make this sustainable, message requests might come from friends themselves. In this case, the figure of the human facilitator could be removed by adding to the system features that include direct communication between participants, supported by algorithms processing an ever-growing context-based (eg, time and location) list of conversational topics and media sharing requests.

### Conclusions

The Media Parcels system was an effective approach to promote media sharing of emotional content for the elderly population that participated in this research. Although the system was tested in only 2 trios of users, it was suitable to promote communication and deepen social relations between participants from different generations (as in study 1) or from the same generation (as in study 2). It can be expected that similar results could be generalizable to the elderly population with characteristics comparable with our sample.

In addition, the participants reported to feel motivated to produce and receive personal media content throughout the study. It is possible, though, that the interest in the use of the system could diminish over time because the system is not novel anymore or producing the media is too burdensome. Nevertheless, the social and personal nature of the media exchange could be motivating to keep users engaged in communication, especially if they feel socially isolated. Moreover, it could be easier for users to keep using the system for longer periods of time if the media requests for deep personal content are balanced with lightweight content and if the users are able to create their own threads of conversation. In fact, the use of a system such as Media Parcels is not necessarily intended to be of long-term. The length of use can be determined, for example, by a health care professional facilitator, focusing on shorter interactions between users according to therapeutic goals.

The Media Parcels design presents a novel solution for including older adults in social media sharing by introducing the concept of intimate directed continuous slow media sharing, which is different from the existing online communities. Our trial showed that the parcel metaphor applied to media content was easily understood by the older population, and the supporting computational system was easy enough to be quickly adopted.

As far as future study is concerned, the authors plan to conduct research expanding the number of participants in a group, targeting specifically older people that are classified as lonely or socially disconnected.

## Appendix

Multimedia Appendix 1 Relationship semantic differential scale.

Multimedia Appendix 2 Participants’ evaluation of their relationship toward one another according to RSDS scores.

Multimedia Appendix 3 Participants’ evaluation of their relationship toward one another according to RSDS scores.

## Abbreviations

ESPIM: Experience Sampling and Programmed Intervention Method
RSDS: Relationship Semantic Differential Scale
SUS: System Usability Scale
UEQ: User Experience Questionnaire
