# Supplementary material for: Promoting Social Connection and Deepening Relations Among Older Adults: Design and Qualitative Evaluation of Media Parcels
Source: J Med Internet Res. 2019 Oct 3;21(10):e14112. doi: 10.2196/14112 (PMC6797971; doi:10.2196/14112)
Supplement: Multimedia Appendix 1 [file jmir_v21i10e14112_app1.pdf]

Semantic Differential Scale

Participant:			Date:
☐ PreT	☐ InT	☐ PosT	

I would like to ask you to rate you relationship with each one of your SSP in the following questionnaire. It consists of pairs of contrasting attributes that may apply to how you feel about the relationship. The circles between the attributes represent gradations between the opposites. You can express your agreement with the attributes by ticking the circle that most closely reflects your impression. Example:

Good	○	⊗	○	○	○	○	○	Bad

This response would mean that you rate the relationship as closer to Good than Bad.

Please decide spontaneously and don't think too long about your decision. Sometimes you may not be completely sure about your agreement with a particular attribute, nevertheless, please tick a circle in every line. It is your personal opinion that counts. Please remember: there is no wrong or right answer!

How would you describe your relationship with ___________________?

Good	○	○	○	○	○	○	○	Bad
Close	○	○	○	○	○	○	○	Distant
Cold	○	○	○	○	○	○	○	Warm
Infrequent	○	○	○	○	○	○	○	Frequent
Joyful	○	○	○	○	○	○	○	Gloomy
Unsupportive	○	○	○	○	○	○	○	Supportive
Superficial	○	○	○	○	○	○	○	Deep
Meaningless	○	○	○	○	○	○	○	Meaningful
Connected	○	○	○	○	○	○	○	Disconnected
Thoughtful	○	○	○	○	○	○	○	Thoughtless
Two-sided	○	○	○	○	○	○	○	One-sided
Boring	○	○	○	○	○	○	○	Exciting
Relaxed	○	○	○	○	○	○	○	Tense
Passive	○	○	○	○	○	○	○	Active
Unsatisfying	○	○	○	○	○	○	○	Satisfying
Prone to communication	○	○	○	○	○	○	○	Not prone to communication
