# Supplementary figures and images for: Promoting Social Connection and Deepening Relations Among Older Adults: Design and Qualitative Evaluation of Media Parcels
Source: J Med Internet Res. 2019 Oct 3;21(10):e14112. doi: 10.2196/14112 (PMC6797971; doi:10.2196/14112)

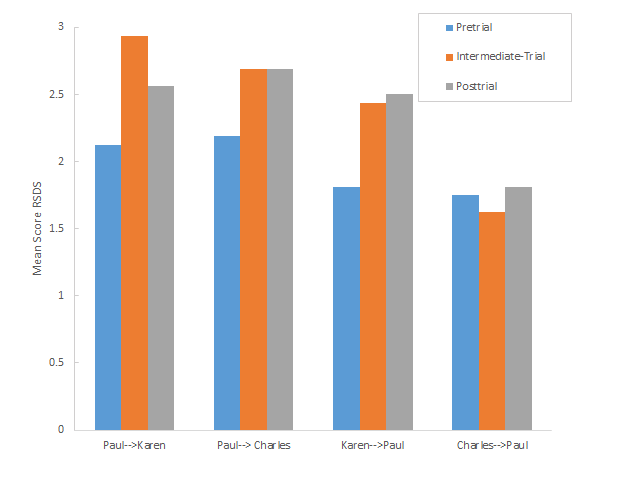

Supplement: Multimedia Appendix 2 [file jmir_v21i10e14112_app2.png]

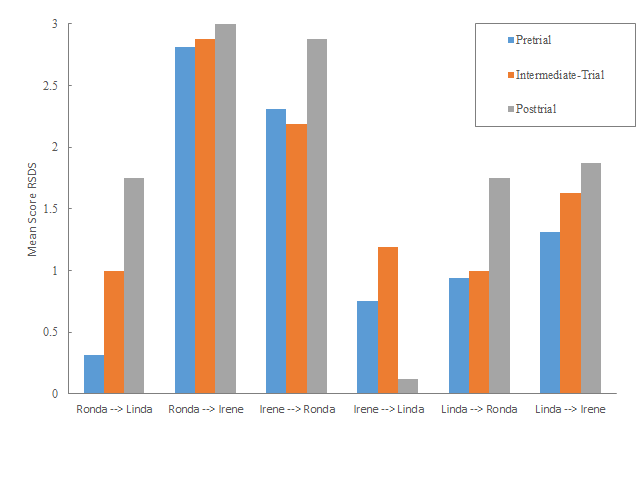

Supplement: Multimedia Appendix 3 [file jmir_v21i10e14112_app3.png]
